# School health promotion and use of drugs among students in Southern Brazil

**DOI:** 10.11606/S1518-8787.2018052000311

**Published:** 2018-05-07

**Authors:** Fernanda Marques Paz, Vanessa Andina Teixeira, Raquel Oliveira Pinto, Cristine Scattolin Andersen, Larissa Prado Fontoura, Luís César de Castro, Marcos Pascoal Pattussi, Rogério Lessa Horta

**Affiliations:** I Universidade do Vale do Rio dos Sinos. Programa de Pós-Graduação em Saúde Coletiva. São Leopoldo, RS, Brasil; II Centro Universitário Ritter dos Reis. Porto Alegre, RS, Brasil; III Instituto Federal de Educação, Ciência e Tecnologia Farroupilha. Farroupilha, RS, Brasil; IV Universidade Federal de Ciências da Saúde. Porto Alegre, RS, Brasil; V Universidade Integrada Vale do Taquari Ensino Superior. Taquari, RS, Brasil; VI Universidade do Vale do Rio dos Sinos. Unidade de Pesquisa e Pós-Graduação. Programa de Pós-Graduação em Saúde Coletiva. São Leopoldo, RS, Brasil; VII Secretária Municipal da Saúde. Centro de Atenção Psicossocial - Álcool e Drogas. Taquara, RS, Brasil

**Keywords:** Adolescent Behavior, Tobacco Use Disorder, epidemiology, Substance-Related Disorders, Alcohol-Related Disorders, Socioeconomic Factors, Health Promotion, Comportamento do Adolescente, Tabagismo, epidemiologia, Transtornos Relacionados ao Uso de Substâncias, Transtornos Relacionados ao Uso de Álcool, Fatores Socioeconômicos, Promoção da Saúde

## Abstract

**OBJECTIVE:**

To analyze the relationship between the health promotion conditions in schools and the consumption of alcohol and other drugs by students.

**METHODS:**

This is a cross-sectional study with a probabilistic sample of 3,464 students aged 12 to 17 from all schools of the cities of Lajeado and Sapiranga, state of Rio Grande do Sul, Brazil, and 53 managers from the same schools; the data was collected in 2012. Reports of the use of tobacco, alcohol, and illicit drugs in 2012 were used as outcomes, and the health promotion score in the school environment was used as the exposure of interest. We submitted the data to multilevel analysis.

**RESULTS:**

The prevalence of the annual use of tobacco was 9.8% (95%CI 8.8-10.8), alcohol was 46.2% (95%CI 44.5-47.8), and other drugs was 10.9% (95%CI 9.9-12.0). In the crude analysis, only the use of tobacco was associated with less health promoting schools (OR = 1.89, 95%CI 1.16-3.09) when compared to those with better conditions. This association lost statistical significance in the adjusted analysis (OR = 1.27, 95%CI 0.74-2.19).

**CONCLUSIONS:**

The effects of the school environment on the use of drugs, especially tobacco and alcohol, are manifested mainly by the individual and family conditions of the adolescents.

## RESUMO

**OBJETIVO:**

Analisar a relação entre condições de promoção de saúde nas escolas e o consumo de álcool e outras drogas pelos escolares.

**MÉTODOS:**

Estudo transversal de base escolar, com amostra probabilística de 3.464 escolares de 12 a 17 anos de todas as escolas de Lajeado e Sapiranga, RS, e 53 gestores das mesmas escolas; dados coletados em 2012. Os relatos do uso de 2012 de tabaco, álcool e drogas ilícitas foram tomados como desfechos e o escore de promoção de saúde no ambiente das escolas, como exposição de interesse. Os dados foram submetidos à análise multinível.

**RESULTADOS:**

A prevalência do uso anual de tabaco foi 9,8% (IC95% 8,8-10,8), álcool 46,2% (IC95% 44,5-47,8) e outras drogas 10,9% (IC95% 9,9-12,0). Na análise bruta, apenas o uso de tabaco esteve associado a escolas menos promotoras (OR = 1,89; IC95% 1,16-3,09) quando comparado as com melhores condições. Essa associação perdeu significância estatística na análise ajustada (OR = 1,27; IC95% 0,74-2,19).

**CONCLUSÕES:**

Os efeitos do ambiente escolar quanto ao uso de drogas, especialmente tabaco e álcool, manifestam-se principalmente pelas condições individuais e familiares dos adolescentes.

## INTRODUCTION

Adolescence is a period marked by many transitions, greater autonomy from parents, and new relationships with friends and at school. Risk behaviors may occur, such as early experiences with alcohol and other drugs^1^ ^,^ ^2^. The magnitude of the occurrence of this type of behavior in Brazil is shown in epidemiological studies^10^ ^,^ ^11^ and justifies the investment in new studies. National surveys on the use of drugs by students in 2004 and 2010 carried out by the Brazilian Center for Information on Psychotropic Drugs (CEBRID) showed an increase in the use of illicit drugs (p ≤ 0.05) and worrisome prevalence at an early age^13^ ^,^ ^17^. The opposite trend was identified for alcohol and tobacco^22^. The National Survey of the Health of Students (PeNSE) of 2012 interviewed students in ninth grade from public and private schools and found a prevalence of 7.3% for the use of illicit substances in life, 19.6% for tobacco, and 66.6% for alcohol^12^ ^,^ ^19^. The main conditions to prevent the use of drugs by students appear in the scope of health promotion actions in a broad sense. Actions to prevent these behaviors stand out among the specific items of health promotion in schools^27^.

The characteristics of school environments, such as physical structure, curricular aspects, and relationships among students are considered capable of influencing student behavior in relation to the use of drugs^4^ ^,^ ^9^. Health promotion actions in schools are discussed worldwide, which prioritize the autonomy of students and minimize possible aggravating factors^8^ ^,^ ^16^. A lower prevalence of the use of alcohol and tobacco is reported in schools with health promotion programs^5^. More welcoming school environments, for example, where students reported better relationships with peers and teachers, have been shown to be associated with a lower prevalence of the use of marijuana^10^. An instrument was recently developed to the evaluate health promotion conditions in schools. It is an instrument adapted to the Brazilian reality and it includes items that refer to the structural characteristics of schools, together with items related to the pedagogical plan and the relationships lived there^21^.

This study aimed to analyze the relationship between the health promotion conditions of school environments and the use of alcohol, tobacco, and other drugs by students.

## METHODS

This is a cross-sectional, school-based study carried out in two medium-sized cities in the state of Rio Grande do Sul, Brazil, in 2012. Fifty-five schools from the cities of Lajeado (n = 33) and Sapiranga (n = 22) participated in the study, and we interviewed students aged 12 to 17 years and the school managers.

Among the students, we carried out probabilistic sampling, preserving proportionality by sex, age, city, and educational network (municipal public, state public, and private). With a single list of classes and respecting the proportionality, we randomly selected 214 classes in the schools of Lajeado and 75 in the schools of Sapiranga. Larger schools had more classes or students. The students received a self-administered questionnaire, developed based on other studies^10^ ^,^ ^11^, pre-coded and standardized for extensive research on the use of psychoactive substances and associated conditions. The questionnaires were deposited in sealed ballot boxes without any personal identification and were only opened at the headquarters of the research group. Interviews were also carried out with school managers, with the use of an instrument to evaluate the health promotion conditions of the school^21^.

In order to ensure the quality of the data collected, previously trained researchers attended the collection in person, all the visits to the classes were checked with the school supervisors, and the researchers contacted the managers to control the data collected. The instruments were entered into the program Epidata 3.5 for later checking and correction.

In addition, the questionnaires of the students contained three pairs of questions that were repeated at different points in the instrument. Different answers to any of these pairs were considered an indication of inattention or disagreement with the filling, being them excluded. We carried out 3,915 interviews and 368 questionnaires (9.4%) were eliminated because they presented inconsistencies or because of the criterion of the duplicated questions, which left us with 3,547 valid interviews. Since two schools refused to participate in the study, a total of 83 interviews were also lost (2.1%). This study includes data from 3,464 students in 53 schools.

The main focus of this study was the low health promotion scores in schools, obtained by applying the school environment evaluation tool^21^ with the interviews of managers, being divided into three dimensions - structural, pedagogical, and relational - and the total score of the instrument. The applied questionnaire had 28 questions, being 20 of them answered by the school manager or his or her representative and eight by direct observation of the interviewer. It is an instrument developed with a focus on the Brazilian reality, with acceptable validity and reliability, with good factor loads (> 0.4), and Cronbach's Alpha above 0.6^21^.

Each dimension of the questionnaire had factors that were defined by the articulation of different items:

- Pedagogical dimension: it involves the subject related to the learning process, analyzing healthy environments. The following items were considered: healthy eating, physical activity, personal hygiene care, sexual and reproductive health, prevention of the use of psychoactive substances, culture of peace, human rights, and personal skills.
- Structural dimension: it contemplates the physical area of the school and its adequacy. This dimension includes the relationship with the community and the partnerships that expanded the promotion and prevention of diseases. Questions about the social environment of the school and rules about rights and duties and events of violence among/in the school community were also addressed.
- Relational dimension: it evaluates the ethos promoting a pleasant environment, from the social point of view. It was observed the occurrence or not of violence, and the relationship between students, teachers, and the community.

The items of the questionnaire were dichotomous variables, with yes or no answers. For the sum of the scores, the answers ‘Yes’ were computed as one point and ‘No’ as zero. In the relational dimension, the following questions had inverted score, that is, when the behaviors did not occur, it was counted as one: “Evidence of physical damage to the school, such as graffiti, depredations”, “In the last 30 school days, did episodes of fights or arguments occur?”, “In the last 30 school days, did verbal assaults occur in the environment?”, and “In the last 30 school days, did verbal aggressions occur in the school environment between students and teachers?”

The total score of each school could vary between zero and 28 points and the contextual variables (structural dimension, relational dimension, pedagogical dimension, and total health promotion score) were integrated into the individual database. The data of each exposure were standardized from zero to 100 using the following formula: (observed value - minimum value / minimum value - maximum value) × 100. They were then categorized according to their quartiles into: 25% less health promoting or less favorable, 50% intermediate, and 25% more health promoting or more favorable. The cut-off points for each dimension were: structural dimension (favorable ≥ 79, moderate 35-78, and unfavorable ≤ 34), relational dimension (favorable ≥ 85, moderate 51-84, and unfavorable ≤ 50), pedagogical dimension (favorable ≥ 94, moderate 48-93, and unfavorable ≤ 47) and, total health promotion score (favorable ≥ 81.1, moderate 52.1-81, and unfavorable ≤ 52).

Outcomes were: use of tobacco, use of alcoholic beverages, and use of other drugs (marijuana, cocaine, ecstasy, or solvents) in the last 12 months. They were obtained using questions with dichotomous answers (yes, no): “Have you smoked in the last year?”, “Have you had any alcoholic beverage in the last year?”, or “Have you used (various descriptions of the substances under study were presented) in the last year”. The questions regarding the use of marijuana, cocaine, ecstasy, and solvents were grouped, and when the use of at least one of these substances was mentioned, we considered it as a positive response. Since the use and marketing of these substances in Brazil is classified as illicit, this outcome was always referred to as the use of illicit drugs in 2012.

The following variables were considered as potential confounding ones: sex^1^ ^,^ ^26^, age group^5^ ^,^ ^10^ (12-13, 14-15, and 16-17 years), education level of the person responsible for the household^1^ ^,^ ^26^ (illiterate or up to fourth grade, from fifth to seventh grade, complete elementary school, complete high school, complete higher education), supervision of the use of the Internet^1^ (with control, does not use the computer, uses and shows sometimes, and uses without control), perception of the relationship with parents^2^ ^,^ ^3^ (good or great with both, fair or poor with one, fair or poor with both), use of tobacco by parents^12^ ^,^ ^13^ (no use of tobacco by parents, father or mother smokes, father and mother smoke, former smokers), use of alcohol by parents^12^ ^,^ ^13^ (no use of alcohol by parents, father or mother: occasional use, father or mother: frequent use, both: frequent use), discrepancy between age and grade^2^ ^,^ ^23^ (yes, no), and psychiatric morbidity (SRQ-20) (yes ≥ 7, no ≤ 6)^7^ ^,^ ^8^.

The description of the variables and their prevalence was carried out in SPSS 22.0. The associations between outcome, exposure, and the other variables were analyzed by Pearson's chi-square test. The effect of the estimated sampling design for the prevalence of the use of tobacco in the year was 4.51, for the use of alcohol it was 12.45, and for the use of illicit drugs it was 2.57. Therefore, subsequent analyses considered the complex nature of the sample. Gross and adjusted odds ratios with their respective 95% confidence intervals (95%CI) were estimated using multilevel logistic regression with students nested into schools using the software MLwiN 2.35.

The multilevel analysis aims to evaluate the effect of contextual variables and individual level variables^24^ ^,^ ^25^. The following steps were independently adopted for each of the outcomes. We started with the creation of the empty model, that is, with only the outcome. The empty model is useful because it allows us to estimate the correlation between levels, correcting the standard errors and the confidence intervals. The outcome was then individually tested with each exposure. Subsequently, individual confounding factors were added. To be considered as a confounding factor, the independent variable should be associated (p < 0.05) with exposure and outcomes. In this sense, in the final models, the outcome of the use of alcohol was controlled for age, sex, education level of the family member, Internet control, relationship with parents, use of tobacco by parents, use of alcohol by parents, discrepancy between age and grade, and Self-Reporting Questionnaire (SRQ-20), while the use of tobacco was adjusted for all the variables reported above, except for education level of the family member and sex (Table 1). The use of illicit drugs was not associated with any of the exposures and their estimates are therefore not presented.

**Table 1:** P-values of Pearson's chi-square test or test for linear trend for the associations between individual independent variables and exposures and outcomes that underwent multiple analysis. Lajeado and Sapiranga, state of Rio Grande do Sul, Brazil, 2012.

Individual independent variable	Use of alcohol in the year	Use of tobacco in the year	Unfavorable Structural Dimension	Unfavorable Pedagogical Dimension	Unfavorable Relational Dimension	Total unfavorable score
Low education level	< 0.001	0.253	< 0.001	< 0.001	< 0.001	< 0.001
Sex: female	0.002	0.295	0.322	0.924	0.993	0.837
Age: 16-17 years	< 0.001	< 0.001	< 0.001	< 0.001	< 0.001	< 0.001
Family does not control Internet use	< 0.001	< 0.001	< 0.001	< 0.001	0.910	< 0.001
Negative relationship with parents	< 0.001	< 0.001	< 0.001	0.018	0.05	< 0.001
Father and mother smoke	< 0.001	< 0.001	0.002	0.004	0.025	< 0.001
Father, mother, or both often drink	< 0.001	< 0.001	< 0.001	0.019	< 0.001	< 0.001
Discrepancy between age and school grade	< 0.001	< 0.001	< 0.001	0.013	< 0.001	< 0.001
7 points or more in the SRQ	< 0.001	< 0.001	0.001	0.022	0.014	< 0.001

SRQ: Self-Reporting Questionnaire

The study was submitted to the Ethics and Research Committee of the Universidade do Vale do Rio dos Sinos in three subprojects, all approved according to Opinions 074/2011 (municipality of Lajeado), 028/2012 (municipality of Sapiranga), and 025/2013 (directors of the schools of Lajeado and Sapiranga, RS, Brazil). The managers of the municipal and state networks and the directors of the private schools signed the consent terms indicating agreement to participate in the study. The informed consent was signed by the guardians of the students.

## RESULTS

The prevalence of the annual use of tobacco was 9.8% (95%CI 8.8-10.8), alcohol was 46.2% (95%CI 44.5-47.8), and illicit drugs was 10.9% (95%CI 9.9-12.0). In the crude analysis, we observed a higher prevalence of the use of alcohol, tobacco, and illicit drugs in students who reported using the Internet without parental supervision, who considered negative the relationship with their parents, who reported being children of alcohol and tobacco users, those with a history of school failure, and those with a positive score for psychiatric morbidity in SQR-20. The students who reported higher education levels for their guardians also had a higher prevalence of alcohol consumption (Table 2).

**Table 2:** Sample distribution and prevalence of use in the year of the substances under study according to individual independent variables. Lajeado and Sapiranga, state of Rio Grande do Sul, Brazil, 2012. (n = 3,464)

Individual variable		n	% use of tobacco	p	% use of alcohol	p	% use of illicit drugs	p
Sex				0.396		0.032		0.624
	Male	1,586	10.3		43.2		10.7	
	Female	1,878	9.5		48.2		11.2	
Age (years)				< 0.001		< 0.001		< 0.001
	12-13	1,508	4.5		24.5		7.8	
	14-15	1,168	11.5		55.5		11.3	
	16-17	788	17.6		73.5		16.5	
Education level of the family member				0.720		0.023		0.070
	Complete high school	1,294	9.6		50.4		10.6	
	Complete elementary school	595	9.9		46.9		9.8	
	Between the 5th and 7th grade	1,044	10.2		43.2		10.4	
	Illiterate or up to 4th grade	398	11.6		43.0		12.4	
Reports monitoring of Internet use				< 0.001		< 0.001		< 0.001
	Uses with control	432	2.1		24.8		3.8	
	Does not use the computer	528	10.8		35.8		10.0	
	Uses and shows sometimes	1,174	5.3		44.5		7.8	
	Uses without control	1,291	16.4		59.1		16.6	
How do you see your relationship with your parents?				< 0.001		< 0.001		< 0.001
	Good or great with both	2,532	7.3		43.0		9.0	
	Fair or poor with one of them	730	16.2		53.0		15.0	
	Fair or poor with both	135	22.2		66.7		21.5	
Use of tobacco by the parents				< 0.001		< 0.001		< 0.001
	No use	1,650	5.9		41.6		7.8	
	Father or mother smokes	553	13.7		49.2		13.2	
	Father and mother smoke	199	15.6		50.3		15.3	
	Former smokers	952	13.9		53.4		13.9	
Use of alcohol by the parents				< 0.001		< 0.001		< 0.001
	No use	925	7.5		38.2		7.6	
	Father or mother: occasional use	956	9.8		44.6		10.4	
	Father or mother: frequent use	1,150	8.8		54.0		10.8	
	Both: frequent use	344	18.3		61.3		20.7	
Discrepancy age-grade				< 0.001		0.001		0.001
	No	3,058	8.7		45.2		10.3	
	Yes	310	20.3		57.1		17.0	
Psychic morbidity (SRQ-20)				< 0.001		< 0.001		< 0.001
	No	2,285	7.3		41.2		8.3	
	Yes	1,039	15.3		57.9		17.0	

SRQ: Self-Reporting Questionnaire Controlled analyses for design effect.

Higher prevalence of the use of tobacco was associated with less health promoting schools in all dimensions and in the total score of the instrument. The use of alcohol was associated with schools with lower relational dimension scores and total scores. The use of illicit substances was not associated with any dimension of the instrument and it was also not associated with the total score (Table 3).

**Table 3:** Absolute (n) and relative (%) distribution of the sample and prevalence of the use of the substances under study according to dimensions and total score in the instrument for the evaluation of the health promotion conditions in schools. Lajeado and Sapiranga, state of Rio Grande do Sul, Brazil, 2012. (n = 3,464)

Variable		Sample		Prevalence of use in the year					
		n	%	Tobacco		Alcohol		Illicit drugs	
				%	p	%	p	%	p
Structural Dimension^a^					**0.011**		0.328		0.059
	Favorable	756	21.8	6.7		41.3		7.9	
	Moderate	1,835	53.0	10.1		46.6		11.4	
	Unfavorable	873	25.2	12.1		48.3		12.1	
Pedagogical Dimension^b^					**0.019**		0.127		0.511
	Favorable	856	24.7	6.1		39.4		10.5	
	Moderate	1,680	48.5	10.2		46.4		10.6	
	Unfavorable	928	26.8	11.8		49.2		11.6	
Relational Dimension^c^					**0.005**		**0.006**		0.291
	Favorable	640	18.5	7.1		38.9		9.7	
	Moderate	1,829	52.8	9.8		44.6		11.0	
	Unfavorable	995	28.7	12.6		54.6		11.6	
Total score^d^					**0.001**		**0.035**		0.065
	Favorable	880	25.4	6.5		40.1		9.1	
	Moderate	1,626	46.9	10.0		44.3		10.9	
	Unfavorable	958	27.7	12.7		53.9		12.3	

a It involves the physical resources, the installed capacity, and the adaptation of the spaces for the activities.

b It includes subjects and activities related to the learning process, such as: healthy eating, physical activity, personal hygiene care, sexual and reproductive health, prevention of the use of licit and illicit drugs, culture of peace, and human rights.

c It includes aspects about the relationship between students, teachers, and the community, the occurrence or not of violence, as well as actions to stimulate the protagonism of students and respect for the standards of coexistence.

d It involves the sum of the standardized scores from 0 to 100 of each of the structural, pedagogical, and relational dimensions. Controlled analyses for design effect. Bold values represent statistically significant associations.

The use of tobacco remained strongly associated with less health promoting schools (total score) and lower scores only in the relational dimension after multilevel analysis (Table 4). The use of alcohol was associated with lower scores only in the pedagogical dimension. These effects were significantly attenuated and lost statistical significance after the control for potential confounding factors at the individual level (Table 5).

**Table 4:** Multilevel analysis with crude and adjusted odds ratios (OR) for use of tobacco in the year according to dimensions and total score in the instrument for the evaluation of the health promotion conditions in schools and individual variables. Lajeado and Sapiranga, state of Rio Grande do Sul, Brazil, 2012. (n = 3,464)

Contextual variable		Use of tobacco in the year			
		Crude analysis		Adjusted analysis^a^	
		OR	95%CI	OR	95%CI
Structural Dimension^c^					
	Favorable	1		1	
	Moderate	1.22	0.77-1.94	1.08	0.71-1.65
	Unfavorable	1.57	0.93-2.65	1.21	0.76-1.94
Relational Dimension^d^					
	Favorable	1		1	
	Moderate	1.53	0.92-2.52	1.49	0.96-2.32
	Unfavorable	**1.90**	**1.10-3.27**	1.59	0.99-2.55
Pedagogical Dimension^e^					
	Favorable	1		1	
	Moderate	1.15	0.75-1.77	1.07	0.72-1.58
	Unfavorable	1.63	0.99-2.70	1.18	0.75-1.85
Total score^f^					
	Favorable	1		1	
	Moderate	1.33	0.87-2.04	1.15	0.77-1.72
	Unfavorable	**1.89**	**1.16-3.09**	1.27	0.74-2.19
Individual variable		Crude analysis		Adjusted analysis^b^	
Age (years)					
	12-13	1		1	
	14-15	**2.75**	**2.02-3.74**	**2.03**	**1.43-2.88**
	16-17	**4.26**	**3.08-5.9**	**2.69**	**1.83-3.94**
Reports monitoring of Internet use					
	Uses with control	1		1	
	Does not use the computer	**5.20**	**2.58-10.47**	**2.78**	**1.31-5.9**
	Uses and shows sometimes	**2.32**	**1.17-4.61**	**1.69**	**0.81-3.53**
	Uses without control	**7.52**	**3.9-14.51**	**4.72**	**2.34-9.50**
How do you see your relationship with your parents?				
	Good or great with both	1		1	
	Fair or poor with one of them	**2.49**	**1.94-3.21**	**1.91**	**1.44-2.54**
	Fair or poor with both	**3.59**	**2.3-5.59**	**2.56**	**1.53-4.28**
Use of tobacco by the parents					
	No use	1		1	
	Father or mother smokes	**2.37**	**1.72-3.28**	**1.94**	**1.35-2.79**
	Father and mother smoke	**2.94**	**1.89-4.59**	**2.00**	**1.21-3.32**
	Former smokers	**2.42**	**1.83-3.2**	**2.09**	**1.53-2.85**
Use of alcohol by the parents					
	No use	1		1	
	Father or mother: occasional use	1.37	0.98-1.91	1.14	0.79-1.64
	Father or mother: frequent use	1.21	0.87-1.68	1.39	0.97-1.99
	Both: frequent use	**2.69**	**1.85-3.92**	**1.95**	**1.28-2.95**
Discrepancy Age-Grade					
	No	1		1	
	Yes	**2.69**	**1.95-3.72**	**1.44**	**1.00-2.08**
Psychic Morbidity (SRQ-20)					
	No	1		1	
	Yes	**2.18**	**1.72-2.75**	**1.68**	**1.29-2.18**

SRQ: Self-Reporting Questionnaire

a Adjusted for age, Internet control, relationship with parents, use of tobacco by parents, use of alcohol by parents, discrepancy between age and grade, and SRQ-20.

b Adjusted between themselves and total health promotion score.

c It involves the physical resources, the installed capacity, and the adaptation of the spaces for the activities.

d It includes subjects and activities related to the learning process, such as: healthy eating, physical activity, personal hygiene care, sexual and reproductive health, prevention of the use of licit and illicit drugs, culture of peace, and human rights.

e It includes aspects about the relationship between students, teachers, and the community, the occurrence or not of violence, as well as actions to stimulate the protagonism of students and respect for the standards of coexistence.

f It involves the sum of the standardized scores from 0 to 100 of each of the structural, pedagogical, and relational dimensions. Bold values represent statistically significant associations (p < 0.05).

**Table 5:** Multilevel analysis with crude and adjusted odds ratios (OR) for use of alcohol in the year according to dimensions and total score in the instrument for the evaluation of the health promotion conditions in schools and individual variables. Lajeado and Sapiranga, state of Rio Grande do Sul, Brazil, 2012. (n = 3,464)

Contextual variables		Use of alcohol in the year			
		Crude analysis		Adjusted analysis^a^	
		OR	95%CI	OR	95%CI
Structural Dimension^c^					
	Favorable	1		1	
	Moderate	1.01	0.63-1.64	1.00	0.78-1.27
	Unfavorable	1.01	0.58-1.77	0.90	0.68-1.20
Relational Dimension^d^					
	Favorable	1		1	
	Moderate	1.04	0.64-1.70	1.09	0.86-1.37
	Unfavorable	1.35	0.78-2.32	1.25	0.97-1.62
Pedagogical Dimension^e^					
	Favorable	1		1	
	Moderate	1.01	0.67-1.51	0.86	0.69-1.08
	Unfavorable	1.72	1.01-2.93	0.97	0.75-1.26
Total score^f^					
	Favorable	1		1	
	Moderate	1.06	0.69-1.61	1.06	0.85-1.33
	Unfavorable	1.56	0.92-2.64	1.12	0.86-1.44
Individual variables		Crude analysis		Adjusted analysis^b^	
Sex					
	Male	1			
	Female	**1.22**	**1.06-1.40**	**1.20**	**1.01-1.44**
Age (years)					
	12-13	1		1	
	14-15	**2.75**	**2.02-3.74**	**3.70**	**3.06-4.49**
	16-17	**4.26**	**3.08-5.901**	**8.84**	**6.88-11.36**
Education level of the family member					
	Complete high school	1		1	
	Complete elementary school	1.06	0.83-1.33	1.13	0.85-1.51
	Between the 5th and 7th grade	1.21	0.92-1.59	1.36	0.99-1.87
	Illiterate or up to 4th grade	**1.32**	**1.03-1.70**	**1.48**	**1.11-1.98**
Reports monitoring of Internet use					
	Uses with control	1		1	
	Does not use the computer	**1.81**	**1.35-2.43**	1.20	0.85-1.69
	Uses and shows sometimes	**2.20**	**1.71-2.83**	**1.75**	**1.30-2.34**
	Uses without control	**3.74**	**2.91-4.81**	**2.59**	**1.94-3.47**
How do you see your relationship with your parents?				
	Good or great with both	1		1	
	Fair or poor with one of them	**1.57**	**1.32-1.87**	**1.34**	**1.09-1.65**
	Fair or poor with both	**2.91**	**1.98-4.28**	**2.62**	**1.66-4.14**
Use of tobacco by the parents					
	No use	1		1	
	Father or mother smokes	**1.42**	**1.16-1.73**	1.18	0.93-1.49
	Father and mother smoke	**1.66**	**1.22-2.25**	1.32	0.92-1.91
	Former smokers	**1.60**	**1.36-1.89**	**1.38**	**1.35-1.41**
Use of alcohol by the parents					
	No use	1		1	
	Father or mother: occasional use	**1.71**	**1.41-2.09**	**1.61**	**1.28-2.02**
	Father or mother: frequent use	**2.51**	**2.07-3.03**	2.77	2.22-3.45
	Both: frequent use	**3.33**	**2.56-4.34**	**2.87**	**2.09-3.92**
Discrepancy Age-Grade					
	No	1		1	
	Yes	**1.79**	**1.39-2.30**	**1.49**	**1.11-2.02**
Psychic Morbidity (SRQ-20)					
	No	1		1	
	Yes	**1.93**	**1.65-2.25**	**1.77**	**1.48-2.13**

SRQ: Self-Reporting Questionnaire

a Adjusted for sex, age, educational level of the family member, Internet control, relationship with parents, use of tobacco by parents, use of alcohol by parents, discrepancy between age and grade, and SRQ-20.

b Adjusted between themselves and total health promotion score.

c It involves the physical resources, the installed capacity, and the adaptation of the spaces for the activities.

d It includes subjects and activities related to the learning process, such as: healthy eating, physical activity, personal hygiene care, sexual and reproductive health, prevention of the use of licit and illicit drugs, culture of peace, and human rights.

e It includes aspects about the relationship between students, teachers, and the community, the occurrence or not of violence, as well as actions to stimulate the protagonism of students and respect for the standards of coexistence.

f It involves the sum of the standardized scores from 0 to 100 of each of the structural, pedagogical, and relational dimensions. Bold values represent statistically significant associations (p < 0.05).

## DISCUSSION

The contextual variables analyzed influence the consumption of substances by students, but the association verified is not kept after adjusting for the variables of the individual level. Environments with better health promotion indicators may present lower prevalence of the use of alcohol and other drugs among students, but only if the effect of the school environment is accompanied by favorable conditions in the family and personal scenarios of the students.

In the crude analysis, the use of tobacco was aggregated to the lowest scores of the relational dimension. One of the items evaluated in this dimension was the relationship between teachers and students. A higher prevalence of the use of tobacco by students is present when these relationships have negative evaluations^16^ ^,^ ^18^. The association between smoking and total scores considered as unfavorable confirmed the findings from other studies^15^ ^,^ ^17^: schools with life skills programs, full-time classes, reinforcement sessions on the use of alcohol and other drugs in the curriculum, better relationships between peers, and higher parental participation in the school showed lower prevalence of the use of tobacco.

Regarding the use of alcohol, we observed an association with lower scores for the pedagogical dimension. According to Malmberget al.^18^, the drinking behavior of young persons is influenced by school standards and by the ability of the institution to provide guidance on the effects of psychoactive substances on the body. Actions such as these approximate the items of the pedagogical dimension grouped in the drug and sexuality factor. In this factor, it is investigated if the school keeps permanent educational actions in its political pedagogical project that stimulate the debate on the risks affiliated to the consumption of alcoholic beverages.

All associations estimated by the crude analysis disappeared when the model was adjusted for individual variables. These variables were divided into: family characteristics (relationship with parents, supervision of Internet use, alcohol and tobacco consumption by parents) and personal characteristics (sex, age, psychiatric morbidity, school performance), which are also described as associated with the use of substances in the current literature^13^ ^,^ ^14^ ^,^ ^26^. Wu et al.^28^ have observed that the influence of school characteristics occurs indirectly via factors related to the family, which may help us understand what was verified in this analysis. Simões et al.^24^ have also shown that social factors are mediated by individual factors in the outcome of the use of drugs.

The results of the association between the total health promotion score and the use of tobacco, as well as the association between the relational dimension and the use of alcohol in the crude analysis, show that the qualification of the environment according to the health promotion guidelines may prevent the use of these substances. However, this may not be enough if taken alone. Specific actions such as full-time classes or counseling and promotion of social skills are indicated. In the United States, a systematic review^6^ has pointed out that the most used strategies in the country context to control and reduce the use of drugs involved training of personal skills and specific actions in programs called universal prevention (for all students), selective prevention (for risk groups), and indicated prevention (students already with problems). Fazel et al.^8^ have shown that schools with mental health services had lower prevalence of the use of alcohol, tobacco, and illicit substances, thus reinforcing the idea that the prevention of these behaviors would depend on more direct and specific actions. The Health in School Program in Brazil provides for collaboration between the school and the community using the Family Health Strategy^20^. There is a challenge for teams from both institutions to strengthen ties and establish partnerships with collaborative action in schools.

This study has a cross-sectional design and therefore does not allow us to examine whether the use of drugs would have determined changes in the health promotion conditions of schools. Health promoting schools could have developed more promotion actions precisely because they have already identified problems related to these behaviors among their students. Another limitation is the lack of power at the contextual level, that is, even though this is a study that covers all schools in the municipalities of Lajeado and Sapiranga, with only two schools refusing to participate, this number of schools may be considered small for multilevel analyses, and the possibility of type II error cannot be completely discarded. In addition, the instrument estimates health promotion conditions in a broad sense. It is not selective for items or characteristics directly related to the use of drugs. However, we have grounds to think that the best health promotion conditions contribute to prevent or delay the contact of young persons with psychoactive substances.

Better health promotion conditions in the school environment may contribute to lower the prevalence of the use of alcohol and tobacco, although only health promotion conditions in the school environment cannot explain drug use by students. Individual or familial factors^24^ ^,^ ^28^ seem to explain substance use better than the contextual characteristics of the school. Other studies are needed to deepen the examination of this question and check alternative possibilities for the relationship between school health promotion and the behavior of students in relation to alcoholic beverages and other drugs.

The effects of the school environment on the use of drugs, especially tobacco and alcohol, are manifested mainly by the individual and family conditions of the adolescents.
